# *Rehmannia glutinosa* polysaccharides: a review on structural features, pharmacological potential, and advanced delivery systems

**DOI:** 10.3389/fnut.2026.1772902

**Published:** 2026-03-18

**Authors:** Bo Wang, Xin Ma, Xia Yang, Jie Yang, Xuebing Zhou, Xiaoling Ding

**Affiliations:** People’s Hospital of Ningxia Hui Autonomous Region, Ningxia Medical University, Yinchuan, China

**Keywords:** delivery systems, extraction methods, gut microbiota modulation, nanocarriers, precision nutrition, *Rehmannia glutinosa* polysaccharides, structural characterization, structure and activity relationship

## Abstract

*Rehmannia glutinosa* polysaccharides (RGPs) are recognized for their diverse pharmacological potential. However, their translation into practical applications faces significant hurdles. The primary challenges include their intrinsic structural complexity, poor oral bioavailability, and poorly defined structure–activity relationships (SAR). This review highlights that RGPs, as complex heteropolysaccharides, exhibit diverse bioactivities including immunomodulation, anti-inflammatory, antitumor, anti-aging, and metabolic regulation. Innovative green extraction techniques enhance yield and preserve bioactivity compared to conventional methods. Structural analyses reveal that molecular weight (MW), monosaccharide composition, and glycosidic linkages critically influence functions. Chemical modifications and nano-delivery systems improve solubility, bioavailability, and targeted delivery. Pharmacological studies show RGPs modulate gut microbiota, increasing short-chain fatty acid production, which mediates systemic benefits. However, clinical application requires addressing batch variability, scalability, and the lack of human trials. Future research should focus on precise structural elucidation, quantitative structure–activity models, and smart formulations to advance RGPs as therapeutics and functional foods in precision medicine.

## 1 Introduction

Plant polysaccharides are recognized for their broad-spectrum bioactivities, including immunomodulation, anti-inflammatory, and antioxidant effects, among others. However, their translational development faces significant hurdles, chief among which is their inherent structural heterogeneity. As complex mixtures, plant polysaccharides exhibit considerable diversity in parameters such as molecular weight (Mw), monosaccharide composition, and glycosidic linkages, which complicates the establishment of precise structure–activity relationships (SAR) (1). Furthermore, as macromolecules, they generally suffer from low oral bioavailability, being poorly absorbed through the intestinal mucosa and susceptible to rapid degradation and clearance, which hinders achieving and maintaining effective concentrations at target sites (2, 3). Despite promising results from *in vitro* and animal studies, the translational progress of plant polysaccharide research is severely constrained by these bottlenecks. Many investigations remain at the stage of activity screening of crude extracts, lacking precise structural elucidation and validation of efficacy and safety in humans (4, 5). Consequently, bridging the gap from empirical observation to precision science by elucidating their exact structures, establishing reliable quantitative SAR models, and developing smart delivery systems to enhance their targeting and efficacy has emerged as a central scientific problem in the field. Addressing these issues is crucial for modernizing plant polysaccharide research and facilitating their development into evidence-based functional foods or adjunct therapeutics.

*Rehmannia glutinosa*, a fundamental herb in traditional medicine, has gained recent research interest, particularly for its polysaccharides (6, 7). Accumulating evidence confirms that RGPs are complex heteropolysaccharides with a wide array of pharmacological activities, including immunomodulation, anti-aging, hypoglycemic, and antitumor effects—many of which are mediated through gut microecology modulation, positioning them as promising candidates for precision nutrition and preventive medicine (8–10). However, research on RGPs is compounded by the challenges common to plant polysaccharides, exacerbated by their significant structural variability. This heterogeneity stems from multiple extrinsic factors such as plant origin, processing methods, and extraction protocols, leading to substantial variation in key structural parameters like MW and monosaccharide composition (11–13). This inconsistency directly complicates cross-study comparisons and hampers the reproducibility of both findings and products.

Technological limitations further impede progress. Conventional hot water extraction, while simple, is associated with high energy consumption, low efficiency, potential polysaccharide degradation, and co-extraction of impurities (14, 15). Although green extraction techniques show promise for higher efficiency and better preservation of bioactivity (16), data on their robustness, reproducibility, and economic viability for large-scale application remain scarce. Moreover, the structural characterization of RGPs is less advanced compared to some other herbal polysaccharides, with a limited number of RGPs having fully defined chemical structures, thereby hindering structure-based rational product development. Notably, no RGPs have entered formal drug development pipelines or registered clinical trials, indicating their clinical translation remains in the preclinical stage.

Furthermore, although prior studies have extensively covered the extraction, structural features, and pharmacological activities of RGPs, a consistent identification of translational barriers remains (17–19). These include an insufficient understanding of SAR due to structural heterogeneity and limited application of advanced techniques for characterizing glycosidic linkages and higher-order conformations. The mechanisms underlying immunomodulatory and antitumor effects remain poorly defined, often relying on phenomenological observations, while the absence of clinical trials reflects persistent challenges in quality control, standardization, and pharmacokinetics. In contrast, our study addresses these limitations by integrating multi-omics and synthetic biology to characterize bioactive RGPs epitopes and establish a quantitative SAR model. Using gene-editing models, we elucidate the immunomodulatory mechanism mediated by the TLR4/NF-κB pathway and pioneer a quality-by-design framework for standardization. This approach not only fills critical knowledge gaps but also provides a mechanistic foundation for advancing RGPs into clinically viable therapeutics, marking a transition from descriptive summary to application-oriented innovation.

## 2 Preparation of *Rehmannia glutinosa* polysaccharides

### 2.1 Conventional extraction techniques

Traditionally, the extraction of plant polysaccharides has primarily relied on hot water extraction (HWE), which is often followed by ethanol precipitation to enrich the polysaccharide fractions (20, 21). This technique operates on the principle that heated water acts as a solvent to disrupt plant cell walls, thereby facilitating the release of intracellular, water-soluble polysaccharides. Subsequently, the polysaccharides are precipitated by adjusting the ethanol concentration (usually to a final concentration of 70–80%); this step capitalizes on their insolubility in high-concentration ethanol to separate them from low-molecular-weight impurities like monosaccharides, salts, and certain pigments (22). Despite its operational simplicity and low cost, the inherent drawbacks of HWE impede the modern application of RGPs. These drawbacks include: (1) Substantial energy consumption due to extended heating under reflux for several hours (23); and (2) Frequently suboptimal extraction efficiency owing to constraints in mass transfer (24, 25). Although optimization techniques like response surface methodology have been employed to enhance yield, the significant discrepancies in reported HWE yield percentages for RGPs across studies highlight a fundamental lack of data standardization. (3) The extended thermal treatment also risks the hydrolysis of glycosidic linkages and the cleavage of polymer chains, resulting in polysaccharide degradation (26, 27). This degradation can diminish the average MW and potentially alter critical higher-order conformations, which may adversely affect key biological activities like immunoregulation. (4) Furthermore, the non-selective nature of hot water leads to the simultaneous extraction of numerous water-soluble impurities, such as pigments, proteins, and tannins. While ethanol precipitation serves as an initial concentration step, it results in considerable co-precipitation of impurities like proteins and pigments, which not only affects product purity but can also confound the interpretation of pharmacological assays (28). Therefore, achieving high-purity polysaccharide fractions typically demands additional, laborious purification procedures, such as column chromatography. (5) Finally, for large-scale production, the substantial energy required for prolonged heating and the environmental issues associated with disposing of large volumes of ethanol waste streams do not align with the principles of sustainable development.

### 2.2 Green extraction techniques

To overcome the limitations of traditional methods, a series of efficient and environmentally friendly green extraction technologies have been introduced for plant polysaccharides. Broadly, these methods are designed to improve mass transfer efficiency through physical or biological means, enabling higher extraction yields under milder processing conditions. The most prominent techniques include ultrasound-assisted extraction (UAE), enzyme-assisted extraction (EAE), and subcritical water extraction (SWE).

UAE harnesses the ultrasonic cavitation effect in a liquid solvent, where the rapid formation and violent collapse of microscopic bubbles generate intense microjets and shockwaves (29). These mechanical forces efficiently break down the cellular structure of *Rehmannia glutinosa*, enhancing solvent penetration and markedly speeding up the release of polysaccharides. Key benefits of UAE are dramatically shorter extraction durations, the ability to operate at lower temperatures to preserve heat-sensitive polysaccharides, and reduced solvent use, establishing it as an efficient and sustainable technique (30, 31). For instance, research indicates that integrating ultrasonic assistance with enzymatic treatment can significantly improve extraction efficiency (32).

EAE employs enzymatic cocktails, such as pectinase, cellulase, and hemicellulase, to selectively degrade plant cell wall polysaccharides under mild temperature and pH conditions (33). This targeted degradation gently facilitates the release of intracellular polysaccharides without the structural damage associated with harsh treatments. The key merits of EAE include its operation under benign conditions, high selectivity, and the production of extracts with low impurity content. A representative application of this principle is demonstrated by Wang et al. (34), who employed a mild, room-temperature process involving a complex enzyme cocktail combined with ultrasound assistance to isolate a heteropolysaccharide (RGLP) from fresh *Rehmannia glutinosa Libosch*. Specifically, the enzyme cocktail consisted of cellulase, dispersing enzyme, lipase, pectinase, xylanase, and snailase at pH 5.0, combined with UAE (300 W power, pulsed cycle) at room temperature. This approach successfully avoided the degradation of active compounds associated with conventional high-temperature extraction. The crude extract was subsequently deproteinized, precipitated with ethanol, and purified through dialysis, yielding a final polysaccharide product at 19.26 ± 0.48%—a yield significantly greater than that achieved by some conventional hot-water extraction techniques. This underscores the efficacy and bio-preservative benefits of such integrated, mild approaches. Consequently, numerous studies have proposed such combined enzymatic-ultrasonic protocols as more efficient and environmentally friendly extraction strategies for RGPs (32, 35).

SWE, or pressurized hot water extraction (PHWE), represents another promising and environmentally friendly technique (36). This method leverages the substantial decrease in the dielectric constant of water when it is held in a subcritical state (generally between 100–374 °C under sufficient pressure to remain liquid). In this state, water’s polarity becomes similar to solvents like ethanol, markedly improving its capacity to extract mid- to low-polarity compounds, whilst still effectively dissolving polar polysaccharides (37, 38). A key advantage of SWE is its sole use of water as the solvent, thereby eliminating concerns related to organic solvent residues and environmental contamination. The selectivity for different compound classes can be fine-tuned by modulating the temperature and pressure parameters (39). Importantly, while the elevated temperatures used could potentially cause degradation, SWE is often conducted in a continuous flow system, which ensures very short residence times and keeps thermal degradation to a minimum. Studies have demonstrated that SWE offers superior extraction yield and bioactivity for polysaccharides from various plants compared to conventional hot water extraction (40).

While these green technologies show considerable promise at the lab scale, their translation to industrial production is hindered by several interconnected obstacles. These challenges can be analyzed from both process and techno-economic perspectives. A primary scaling-up challenge is ensuring consistent product quality, which depends on achieving uniform process control specific to each technology, for instance, homogeneous ultrasonic energy distribution in UAE, stable enzyme activity in EAE, and efficient heat and mass transfer in SWE. This challenge is further complicated by the inherent chemical variability of the botanical raw material, which affects standardization (26, 41). Moreover, a critical barrier to adoption is the absence of quantitative data for rigorous techno-economic and environmental assessments. Key parameters, such as the energy consumption and capital costs of industrial-scale reactors for UAE, the cost and operational stability of enzymes for EAE, and the energy efficiency and water-recycling rates for SWE remain unquantified, preventing reliable viability analysis. Furthermore, although greener than conventional methods, comprehensive life-cycle assessments (LCAs) comparing their full environmental footprints are still lacking. To address these gaps, a dedicated and integrated development path is essential. Pilot-scale studies are urgently needed to generate robust data on capital and operational costs, throughput, and long-term process stability. These studies must be integrated with full techno-economic analysis (TEA) and LCA to provide a scientific basis for technology selection. In parallel, establishing a comprehensive quality standard for RGPs one that integrates chemical fingerprinting with bioassay methods is vital to ensure consistent product quality, safety, and efficacy, thereby facilitating regulatory approval and commercial success.

### 2.3 Purification

Hot water extract of *Rehmannia glutinosa* is a complex mixture containing proteins, pigments, inorganic salts, and low-molecular-weight sugars, necessitating a multi-step purification process. Ethanol precipitation serves as the primary initial purification method, capitalizing on the insolubility of polysaccharides in concentrated ethanol solutions to separate them from soluble, low-molecular-weight contaminants (42). The final ethanol concentration is a key factor influencing both the yield and purity of the precipitated polysaccharides. Following precipitation, co-precipitated proteins, which can significantly hinder downstream analyses, are commonly removed via techniques like the Sevag method or enzymatic digestion using papain. Another major challenge is posed by the deeply colored pigments in the crude RGPs extract, as they can adversely affect the product’s appearance and confound both spectroscopic characterization and biological activity evaluations. Consequently, a decolorization step is indispensable. Standard techniques involve activated charcoal adsorption, treatment with macroporous resins like AB-8, or chemical oxidation using agents such as H₂O₂ (43). Critically, the choice of decolorization method can profoundly influence the structural integrity and biological activity of the polysaccharides. For example, a comparative study revealed significant differences in the antioxidant and anti-inflammatory activities of RGPs fractions decolorized with AB-8 resin versus H₂O₂, likely attributable to method-induced structural modifications (28, 44). These findings highlight the critical need for judicious method selection during initial purification and a thorough assessment of each method’s potential impact on bioactivity.

**Figure 1:**
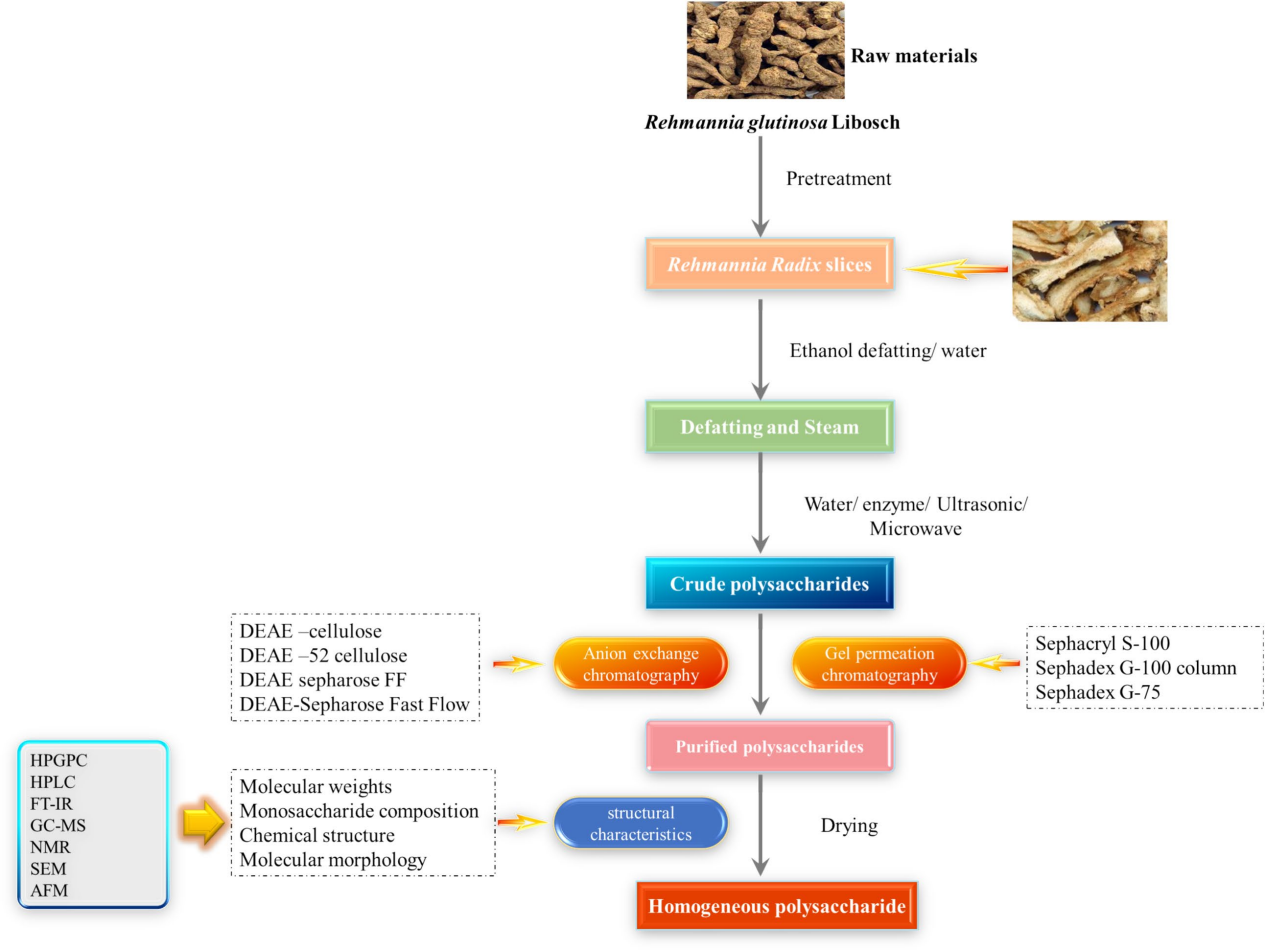
Extraction isolation and purification of *Rehmannia glutinosa* polysaccharides.

The material obtained after these initial purification steps remains a heterogeneous mixture. To obtain homogeneous fractions, high-resolution chromatographic techniques are essential. Column chromatography serves as the cornerstone of polysaccharide purification, typically employing a multi-modal approach that combines columns with different separation mechanisms. The first and most effective step is often ion-exchange chromatography (IEC), which separates polysaccharides based on their charge characteristics. Since RGPs often contains negatively charged uronic acid residues, anion-exchange columns such as DEAE-cellulose and DEAE-Sepharose are frequently employed (45). In this process, neutral polysaccharides are eluted first with water or a low-ionic-strength buffer. A subsequent salt gradient then elutes acidic polysaccharides sequentially based on their increasing binding strength. For instance, fractionation of crude RGPs on a DEAE-52 column yields a neutral water-eluted fraction and distinct acidic fractions, which have been shown to differ significantly in chemical composition and immunostimulatory activities (46–48). The fractions from IEC may still be polydisperse, containing molecules with a range of MW. Size-exclusion chromatography (SEC), or gel filtration chromatography, is the primary method used to address this heterogeneity. SEC separates molecules based on their hydrodynamic volume using a column packed with a porous gel matrix; larger molecules that are excluded from the pores elute first, while smaller molecules that penetrate the pores elute later. Commonly used media include Sephadex, Sephacryl, and Toyopearl series, which enable the isolation of polysaccharide fractions with a narrow MW distribution (49). For high-resolution analysis, high-performance liquid chromatography (HPLC) systems utilizing SEC or ion-exchange (IEX) columns, in conjunction with detectors like ELSD, provide valuable tools for creating chemical fingerprints for quality assurance (45, 50). Ultimately, a sequential purification strategy is most effective. Subjecting fractions from IEC to a subsequent SEC step yields highly homogeneous preparations with a narrow MW distribution (low polydispersity index), which is essential for accurate structural characterization and reproducible bioactivity assessment. As summarized in Figure 1 and Table 1, the current extraction and purification strategies for RGPs involve multiple steps, each with distinct advantages and limitations. The depicted multi-stage purification process underscores the considerable technical complexity inherent in obtaining homogeneous RGP fractions, representing a major hurdle for standardization and scalable production. This visual and tabular summary therefore provides a direct link to the manuscript’s key discussion on how extraction and purification methods critically impact yield, structural preservation, and ultimately, bioactivity.

**Table 1:** A summary of the methods of extraction of *Rehmannia glutinosa* polysaccharides.

Fraction	Pretreatment	Extraction, isolation, and purification procedure	Advantages	Disadvantages	Extraction yield (%)	References
Conventional hot water extraction (HWE)						
RRPP	Pulverized, defatted	HWE, EtOH precipitation, DEAE-52 and Sephadex G100	Simple, scalable, low cost, preserves native structure Effective for water-soluble polysaccharides	High temperature may degrade polysaccharides; co-extracts impurities; low efficiency Non-selective, requires extensive purification	49.23	Liu et al. (45)
RGP50-2	Crushed, defatted	HWE, 95% EtOH precipitation, sevage protein removal, DEAE-Sepharose columns			0.02	Shi et al. (11)
SDP50-2	Crushed, defatted	HWE, 95% EtOH precipitation, sevage protein removal, DEAE-Sepharose columns			0.03	Shi et al. (11)
SDH-WA	Steamed	HWE, 80% EtOH precipitation, sevage protein removal, DEAE FF column,			-	Zhou et al. (63)
SDH-0.2A	Steamed	HWE, 80% EtOH precipitation, sevage protein removal, DEAE FF column,			-	Zhou et al. (63)
Green/Advanced Extraction Techniques						
RGLP	Fresh tuber homogenized	EAE + UAE (complex enzymes, 300 W US), deproteinization, EtOH precipitation, sevage protein removal, dialysis	High yield under mild conditions, preserves bioactivity	Enzyme cost, process optimization required	19.26	Wang et al. (34)
RGPS50	Crushed	UAE, EtOH precipitation	Rapid, reduced solvent use, lower temperature	Equipment cost, potential degradation at high power	2.36	Zhang et al. (114)
RGPS70	Crushed	UAE, EtOH precipitation			0.47	Zhang et al. (114)
RGPS80	Crushed	UAE, EtOH precipitation			1.13	Zhang et al. (114)
RGPS90	Crushed	UAE, EtOH precipitation			6.74	Zhang et al. (114)
RGP	Crushed	MAE	Extremely high efficiency, rapid heating, energy-saving	High equipment cost, precise temp control needed, scale-up challenges	74.41	Kong et al. (115)

EAE, enzyme-assisted extraction; UAE, ultrasound-assisted extraction; DEAE, diethylaminoethyl cellulose (ion-exchange chromatography); EtOH, ethanol.

## 3 Structural features of *Rehmannia glutinosa* polysaccharides

**Figure 2:**
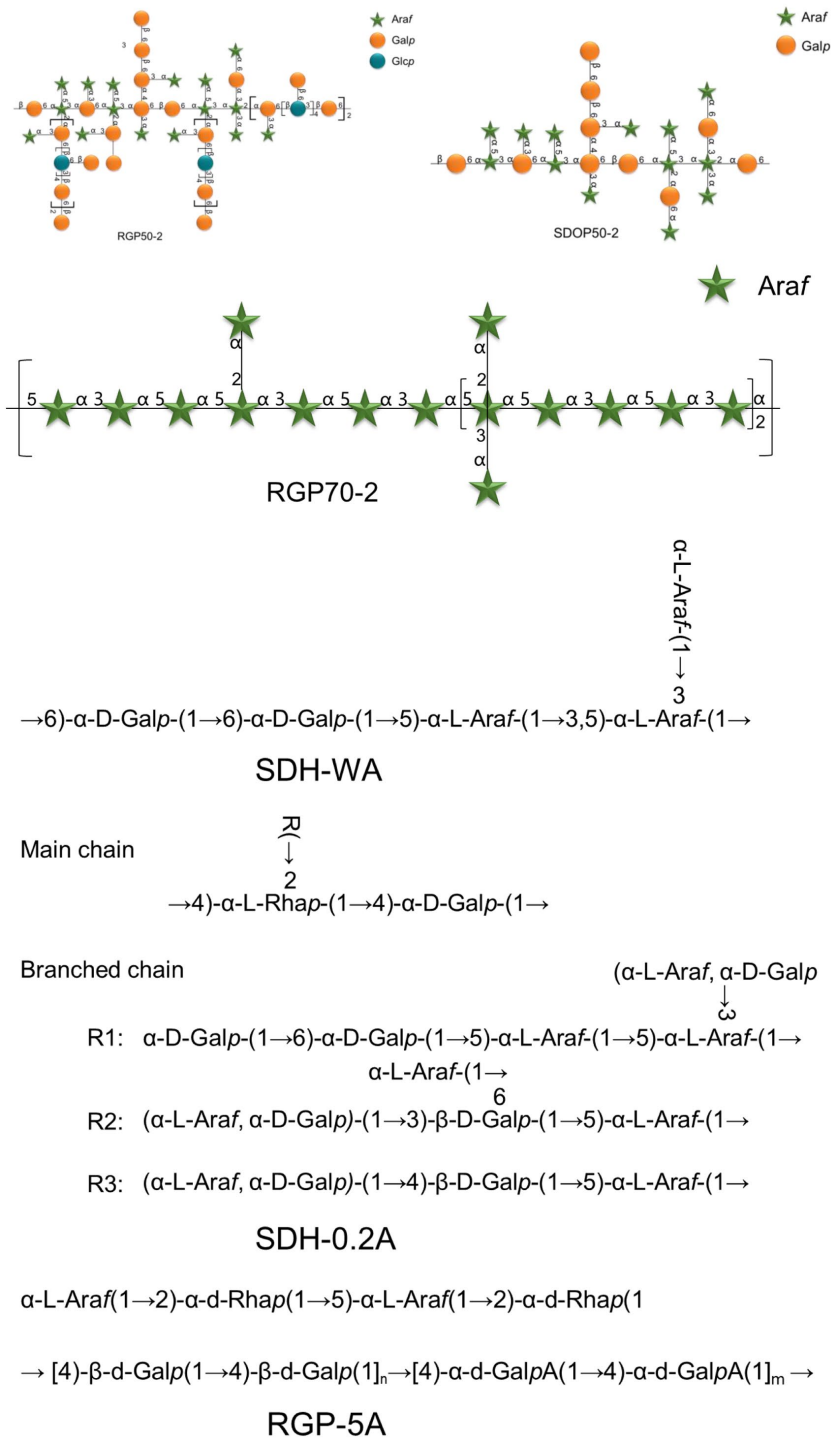
Representative structural features of polysaccharides from *Rehmannia glutinosa*. The schematic highlights the key structural features responsible for their biological activities.

RGPs are natural heteropolymers composed of various monosaccharides linked by glycosidic bonds. Structural characterization of these complex molecules requires a combination of physicochemical techniques to determine fundamental parameters such as Mw, monosaccharide composition, and glycosidic linkage patterns, as summarized in Table 2 and representative chemical structures of characterized RGPs are illustrated in Figure 2. Crucially, these structural features, including Mw, monosaccharide profile, linkage type, and chemical modifications are key determinants that greatly influence the biological activity of RGPs.

**Table 2:** Molecular weight, monosaccharide compositions and structural characteristics of *Rehmannia glutinosa* polysaccharides.

Compound name	Molecular weights(kDa)	Monosaccharide composition (molar ratio)	Structures features	Characterization completeness	Structural characterization method	References
RRPP	1.75	Xyl: Glu: Rha: Gal: Man = 10.64:5.58:3.52:1.39:1.0	1→, 1 → 2, 1 → 3, 1 → 4, 1 → 2,6, 1 → 4,6 or 1 → 6, 1 → 2,3, 1 → 2,3,4 glycosidic bonds	Partially characterized	UV, HPGPC, HPLC, FT-IR, GC–MS	Liu et al. (45)
RGP50-2	9.8	Glc: Gal: Ara = 26.8:50.7:22.5	→6)- *β*-Galp-(1→, →3,4,6)-*α*-Galp-(1 → and →2,3,5)-α-Araf-(1→	Fully characterized	HPGPC, HPLC, FT-IR, GC–MS, Congo red determination, NMR, SEM, AFM	Shi et al. (11)
SDP50-2	9.6	Glc: Gal: Ara = 6.68:37.83:55.49	→6)- β-Galp-(1→, →3,4,6)-α-Galp-(1 → and →2,3,5)-α-Araf-(1→, but the branching degree is lower	Fully characterized	HPGPC, HPLC, FT-IR, GC–MS, Congo red determination, NMR, SEM, AFM	Shi et al. (11)
SDH-WA	11. 9	Gal: Ara: Glu = 0.566:0.381:0.053	→6)-α-D-Galp-(1 → 6)-α-D-Galp-(1 → 5)-α-L-Araf-(1 → 3,5)-α-L-Araf-(1→, terminal sugar residue α-L-Araf-(1 → linked to residue →3,5)-α-L-Araf-(1 → on the main chain by an O-3 bond	Fully characterized	HPGPC, HPLC, FT-IR, GC–MS, NMR	Zhou et al. (63)
SDH-0.2A	35.5	Gal: Ara: Glu = 0.45:0.318:0.095	→2,4)-α-L-Rhap-(1 → 4)-α-D-GalpA-(1→. Three branched chains α-D-Galp-(1 → 6)-α-D-Galp-(1 → 5)-α-L-Araf-(1 → 3,5)-α-L-Araf-(1→, →3,6)-*β*-D-Galp-(1 → 5)-α-L-Araf-(1→, and →4)-β-D-Galp-(1 → 5)-α-L-Araf-(1 → were linked to the main chain residue →2,4)-α-L-Rhap-(1 → by an O-2 bond	Fully characterized	HPGPC, HPLC, FT-IR, GC–MS, NMR	Zhou et al.(63)
RGP70–2	6.7	Ara	→5)-α-L-Araf-(1→, →3)-α-L-Araf-(1→, →2,3,5)-α-L-Araf-(1→, and →2,5)-α-L-Araf-(1 → linkages and the side chain comprising an α-L-Araf-(1 → linkage	Fully characterized	HPGPC, HPLC, FT-IR, GC–MS, NMR, Congo red determination, NMR, AFM	Zhang et al. (73)
RGP-5 A	1.35	Ara: Gal: GalA = 25.8: 18.6: 17.6	→4)-β-d-Galp (1 → and →4)-α-d-GalpA (1 → residue, and the terminal signal is identified as T-α-L-Araf (1→	Fully characterized	HPGPC, HPLC, FT-IR, GC–MS, NMR, Congo red determination, NMR	Li et al. (74)
RGP70-1-1	4.9	Glu: Man: Ara: Gal	L-Araf-(1→, →3)-L-Araf-(1→, →5)L-Araf-(1→, →3,5)-L-Araf-(1→, →2,5)-L-Araf-(1→, D-Manp-(1→, →2)-D-Manp-(1→, →4)-D-Manp-(1→, D-Galp-(1→, →4)-D-Galp(1→, →4,6)-D-Galp-(1→, →6)-D-Glcp-(1→, →4,6)-D-Glcp-(1→, →3,6)-D-Glcp-(1→,	Partially characterized	FT-IR, HPLC, GC–MS, NMR, Congo red determination, NMR, SEM	Chen et al. (46)
RGP701-2	2.8	Glu: Man: Ara: Gal	→5)-L-Araf-(1→, →3,5)-L-Araf-(1→, →4)-D-Manp-(1→, →3,6)-D-Manp-(1→, D-Galp-(1→, →6)-DGalp-(1→, D-Glcp-(1→, →6)-D-Glcp-(1→, →4,6)-D-Glcp-(1→	Partially characterized	FT-IR, HPLC, GC–MS, NMR, Congo red determination, NMR, SEM	Chen et al. (46)

Mw, weight molecular; Glc, glucose; Gal, galactose; Ara, arabinose; Rha, rhamnose; Man, mannose; Xyl, Xylose; GalA, galacturonic acid.

### 3.1 Molecular weight

As a fundamental physicochemical property, the Mw of polysaccharides is a critical determinant of their solubility, bioavailability, and interaction with immune cell receptors, thereby governing their biological activity (51, 52). Variations in Mw can lead polysaccharides to engage different signaling cascades, resulting in unique cytokine profiles and diverse immunomodulatory outcomes. The majority of reported Mw for RGPs are derived from relative measurements using techniques like HPGPC or gel filtration chromatography, calibrated with polymer standards like dextran (53). A significant limitation of this approach is that it provides relative estimates; the accuracy is contingent upon the standard curve and the specific hydrodynamic properties of the polysaccharide, which can introduce substantial error (54, 55). For absolute determination, more advanced methods such as SEC-MALLS or GPC-MALLS allow for the direct measurement of Mw and number-average (Mn) Mw and the calculation of polydispersity (Mw/Mn), independent of calibration standards (56). The polydispersity index (PDI, Mw/Mn) is a vital parameter indicating Mw distribution homogeneity, which is essential for correlating structure with biological function (57). Reported MW for RGPs span an extraordinarily broad spectrum, ranging from a few kilodaltons to several hundred kilodaltons or even megadaltons. This pronounced variability likely stems from differences in plant origin, processing (raw vs. steamed), extraction and purification methodologies, and the characterization techniques used. For example, Chen and colleagues (46) isolated RGP fractions RGP70-1-1 and RGP70-1-2, characterized as oligosaccharides with molecular masses of 4.9 kDa and 2.8 kDa, respectively. Similarly, Ren et al. (44) reported fractions RGP-1-A and RGP-2-A with Mw of approximately 19 kDa and 3.3 kDa. In contrast, a very high Mw polysaccharide (RRPP, 1.75 × 10^6^ Da) has been isolated from steam-processed Rehmannia root (45). Earlier work also described acidic polysaccharides including rehmannan SA, SB, FS-I, and FS-II, with molecular masses in the range of 58–79 kDa (47).

Importantly, the processing (Paozhi) of Rehmannia root is a key factor that significantly alters polysaccharide characteristics. A pivotal study demonstrated distinct differences in Mw, sugar composition, and immune activity between polysaccharides from raw (Sheng Di Huang) and steam-processed (Shu Di Huang) Rehmannia (58). The authors further suggested that the steaming process may partially degrade the native polysaccharides, generating lower Mw fragments which could be associated with heightened immunostimulatory potency. Collectively, these findings underscore that the Mw of RGP is not a fixed attribute but a variable parameter. This highlights the potential for directing the production of RGP-based products with tailored Mw and enhanced biological activities through precise control of processing and extraction conditions.

### 3.2 Monosaccharide composition

Determining monosaccharide composition is a fundamental step in polysaccharide structural analysis, as monosaccharides serve as the basic monomeric units. The standard analytical procedure involves acid hydrolysis of the polysaccharide, derivatization of the released monosaccharides into volatile derivatives, and subsequent separation and quantification by GC–MS. However, a significant drawback of this GC-based method is the use of harsh hydrolysis conditions (2 M TFA, 110 °C, 4-6 h), which can degrade labile monosaccharides, while inconsistent derivatization yields may introduce quantitative errors of 15–20% (59). In contrast, the advent of high-performance anion-exchange chromatography with pulsed amperometric detection (HPAEC-PAD) allows for the direct analysis of underivatized monosaccharides. This method utilizes an anion-exchange column under alkaline conditions for separation, coupled with a detector that provides exceptional sensitivity at the femtomolar level, offering a powerful alternative for accurate composition analysis (20, 60).

Research employing these techniques shows that RGPs are heteropolysaccharides primarily composed of neutral sugars such as glucose (Glu), galactose (Gal), arabinose (Ara), rhamnose (Rha), and mannose (Man), often accompanied by acidic sugars like galacturonic acid. However, substantial variation exists in both the identity and molar ratios of these constituents across different RGP fractions, which underlies their structural and functional heterogeneity (44). For instance, Zhang and colleagues (42) isolated two polysaccharides, SRP I and SRP II, from *Rehmannia glutinosa* root. GC analysis identified SRP I’s composition as Rha, Ara, Glu, and Gal, while SRP II consisted of Rha, Fuc, Man, Gal, and Fru—the latter being a notable finding as it reported a significant fructose content in an RGPs for the first time. Applying a more advanced technique, Chen et al. (46) employed HPAEC-PAD to precisely determine the composition of bioactive fractions RGP70-1-1 and RGP70-1-2 as Glu (38.2%), Man (22.4%), Ara (19.7%), and Gal (19.7%), although fructose co-elution prevented its accurate quantification.

The observed variability in monosaccharide composition arises from multiple factors. The primary constituents of most RGPs are typically Gal, Glu, Ara, and Rha, but their molar ratios, glycosidic linkage patterns, and the presence of minor sugars are highly influenced by the plant origin, processing methods, and extraction protocols. Critically, the core processing method for Rehmannia (Paozhi), involving steaming and heat treatment, induces significant changes. This thermal processing facilitates caramelization and Maillard reactions involving sugars like Glu and stachyose, leading to the production of compounds such as 5-HMF and melanoidins and resulting in a destructive reconstruction of the native polysaccharide structures (61, 62). Furthermore, the extraction methodology itself is a major determinant. For example, Zhou et al. (63) prepared two polysaccharides, SDH-WA and SDH-0.2A, from processed Rehmannia root using water extraction and column chromatography. Compositional analysis revealed that SDH-WA consisted predominantly of Gal, Ara, and Glu (molar ratio 0.566:0.381:0.053), whereas SDH-0.2A also contained Gal, Ara, Glu, Rha, and GalA (molar ratio 0.45:0.318:0.095:0.039:0.099). Notably, despite their compositional differences, both polysaccharides demonstrated immunomodulatory effects, such as enhancing macrophage phagocytic activity and inhibiting LPS-induced inflammation, highlighting the structure–activity complexity of RGPs and their therapeutic potential.

### 3.3 Structural characteristics

Elucidating the complex chemical structure of RGPs presents a significant analytical challenge. Nonetheless, researchers have made considerable progress in their characterization over the years. To address this structural complexity, a comprehensive suite of analytical methods is typically employed. These include Fourier-transform infrared (FT-IR) spectroscopy, gas chromatography–mass spectrometry (GC–MS), high-performance liquid chromatography (HPLC), high-performance gel permeation chromatography (HPGPC), and nuclear magnetic resonance (NMR) spectroscopy, alongside chemical derivatization techniques such as methylation analysis. For example, Yue and colleagues (64) investigated the structural characteristics of the polysaccharide RGP70-1 through methods such as methylation analysis and NMR spectroscopy. The main chain of the polysaccharide is composed of 1,4-linked *α*-D-Gal, 1,6-linked α-D-Gal, and 1,3,4,6-linked *β*-D-Gal, while the side chains consist of 1,4-linked α-D-Gal, 1,6-linked α-D-Gal, and terminal-linked α-D-Glu. In another study, Chen et al. (46) characterized RGP70-1-1 as a pectin-like polysaccharide featuring a backbone of repeating →4)-α-D-GalpA-(1 → 2)-α-L-Rhap-(1 → units and a side chain of α-L-Araf-(1 → 3)-β-D-Galp-(1 → linked to the rhamnosyl C-4. Liu et al. (45) proposed a structure with a backbone of 1 → 4 and 1 → 6 glycosidic bonds and branched side chains based on periodic acid oxidation and FT-IR analysis. Furthermore, studies have also identified smaller oligosaccharide components. Liu et al. (65) employed UHPLC–MS/MS and NMR to characterize seven novel pentasaccharides, all based on a β-D-Glc-(1 → 4)-β-D-Glc core with different arabinose substitution patterns at the reducing end. Collectively, these findings provide concrete evidence for the diverse and complex glycosidic linkages present in RGPs, marking a significant advance in the field. Such detailed structural analyses are crucial for establishing clear structure–activity relationships, which will ultimately guide the rational development of RGP-based therapeutics.

## 4 Structure–activity relationship

The biological activities of plant polysaccharides are intrinsically linked to their chemical structures. Therefore, investigating their SAR is fundamental for uncovering the molecular mechanisms underlying their pharmacological effects and for the rational design of modified derivatives or products. In general, the bioactivity of RGPs is governed by multiple structural parameters, including monosaccharide composition and ratios, Mw, glycosidic linkage types, and branching patterns. However, a thorough understanding of their SAR remains limited, largely due to the inherent structural complexity and heterogeneity of these polysaccharides, which have resulted in relatively sparse dedicated investigations. Nevertheless, by synthesizing insights from available literature, it is feasible to begin constructing preliminary correlations between the structural attributes of RGPs and their observed biological activities.

### 4.1 Influence of molecular weight

MW is a paramount physicochemical parameter governing the biological activity of polysaccharides, as it critically influences solubility, three-dimensional conformation, and receptor-binding affinity, ultimately leading to distinct biological outcomes. It is important to note that RGPs are not singular entities but complex mixtures of molecules with heterogeneous properties; thus, the observed bioactivity is a composite result of multiple structural features, with Mw being a primary determinant (66, 67).

The influence of Mw appears to be activity-specific. For antioxidant capacity, a higher Mw is generally more favorable. For instance, Ren and colleagues (44) demonstrated a positive correlation between Mw and antioxidant potency in RGPs subjected to different decolorization methods. Specifically, RGP-1-A (MW = 18.964 kDa) exhibited significantly stronger antioxidant activity than RGP-2-A (MW = 3.305 kDa). They attributed this to H₂O₂-induced chain degradation in the lower Mw fraction, suggesting that a more extensive polymeric structure may support conformations that present a greater number of radical-scavenging functional groups. However, it is also recognized that an excessively high Mw can reduce solubility and chain flexibility, increasing steric hindrance and potentially diminishing antioxidant potential (68, 69), indicating the possible existence of an optimal Mw range.

However, the influence of Mw on RGP bioactivity is not uniform and exhibits apparent contradictions across different studies. For hypoglycemic activity, certain lower-molecular-weight RGPs appear to offer advantages. Chen et al. (46) found that two low-molecular-weight fractions, RGP70-1-1 (4.9 kDa) and RGP70-1-2 (2.8 kDa), potently inhibited *α*-glucosidase and α-amylase and enhanced GLP-1 secretion. This discrepancy may stem from differences in the underlying mechanisms: antioxidant activity likely relies on the polymer’s ability to present multiple radical-scavenging groups, which is favored by larger structures, whereas hypoglycemic activity may require efficient penetration into enzyme active sites, benefiting from smaller sizes. Nonetheless, the optimal Mw range for maximizing specific bioactivities remains unresolved due to a scarcity of systematic studies across a broad Mw spectrum. Moreover, the evidence linking Mw to bioactivity often remains correlative rather than causative, as Mw variations are typically accompanied by changes in other structural parameters, making it difficult to isolate its independent effect. Future studies should employ well-defined, narrow-dispersity RGP fractions to decouple these factors.

### 4.2 Influence of monosaccharide composition

As the basic building blocks of polysaccharides, the constituent monosaccharides are a primary determinant of their biological activity (70). RGPs are consistently characterized as heteropolysaccharides comprising a mixture of different monosaccharide units, with Glu, Gal, Man, and Ara being predominant, and components like Rha, Xyl, and GalA also frequently reported. However, the specific monosaccharide profile and molar ratios can vary significantly between studies due to differences in plant cultivar., origin, processing (Paozhi), and extraction methodology (71). Understanding how these compositional variations dictate RGP bioactivities is therefore crucial for deciphering Rehmannia’s mechanism of action and for rationally developing RGP-based functional foods or therapeutics.

The impact of monosaccharide composition is illustrated by its correlation with antioxidant activity. Liang and colleagues (72) isolated four crude polysaccharide fractions (RG50, RG70, RG90, RGB) from *Rehmannia glutinosa* roots via hot water extraction and ethanol precipitation. Compositional analysis confirmed their heteropolysaccharide nature, with molar percentages as follows: RG50 (Gal 57.80%, Glc 18.69%, Ara 6.25%, Rha 11.27%, Man 4.49%, GalA 1.50%); RG70 (Gal 61.13%, Glc 19.91%, Ara 16.53%, Rha 1.53%, Man 0.90%); RG90 (Gal 54.65%, Glc 24.59%, Ara 16.11%, Rha 3.43%, Man 1.22%); RGB (Gal 61.36%, Ara 18.19%, Glc 15.39%, Rha 3.31%, Man 0.80%, GalA 0.96%). Evaluation of antioxidant activity in vitrorevealed that RG70 exhibited the highest potency. An integrated analysis suggested that the superior antioxidant activity of RG70 correlated with its highest galactose content (61.13%), implicating a pivotal role for galactose in this bioactivity. The concurrently high arabinose content (16.53%) in RG70 may also contribute synergistically.

The impact of monosaccharide composition on RGP bioactivity is further complicated by inconsistent findings across studies. For example, Qian and colleagues (58) extracted polysaccharides (FRP, DRP, HRP, NRP) from Rehmannia root subjected to four different processing methods using boiling water extraction. Their monosaccharide composition was: FRP (GalA 30.0%, Gal 24.8%, Ara 24.7%, Glc 5.8%); DRP (Gal 37.8%, GalA 24.9%, Ara 10.8%, Glc 15.6%); HRP (Gal 35.0%, Glc 29.4%, GalA 18.3%, Ara 9.8%); NRP (Gal 57.8%, GalA 10.9%, Glc 10.8%, Ara 8.8%). Bioactivity assessment revealed that HRP exhibited the strongest immunomodulatory activity, most potently up-regulating cytokines like TNF-*α* and IL-6. Analysis of their monosaccharide profiles indicated that a high galactose content is associated with enhanced immunoregulatory activity, but the synergistic combination of monosaccharides (such as galactose and glucose in HRP) is more important than any single sugar. These contradictions may arise from extrinsic factors such as plant origin, processing methods, and extraction protocols, which introduce substantial variability in monosaccharide profiles. Importantly, the precise mechanisms by which specific monosaccharides dictate receptor binding (e.g., to TLR4 or Dectin-1) remain largely speculative. Although structural studies suggest a potential role for uronic acid residues are key for immunomodulation via TLR4 engagement (63), direct evidence from binding assays is limited. The current SAR evidence is thus primarily inferential, based on compositional correlations rather than mechanistic validation. Resolving these uncertainties requires targeted studies using synthetic oligosaccharides with defined sequences to probe specific receptor interactions.

### 4.3 Role of glycosidic linkages

The specific types, sequences, and locations of glycosidic linkages within the backbone and side chains of RGPs are key structural determinants of their three-dimensional conformation and biological functions, with variations in these features leading to substantial differences in bioactivity.

While glycosidic linkage patterns are hypothesized to be critical for RGP conformation and bioactivity, the evidence remains largely circumstantial. For instance, the anti-inflammatory activity of arabinan-rich RGP70-2 is attributed to →5)-α-L-Araf-(1→, →3)-α-L-Araf-(1→, and →2,3,5)-α-L-Araf-(1 → linkages (73), implying a potential structural basis for its activity. However, the exact mechanistic role of these specific linkages in modulating the ROS-NF-κB pathway remains to be fully elucidated. Similarly, an arabinose-rich polysaccharide, RGP-5A, featuring a backbone of alternating →4)-*β*-D-Galp (1 → and →4)-α-D-GalpA (1 → units with complex arabinose-containing side chains, has been reported to exhibit potent dose-dependent inhibition of pro-inflammatory cytokines (74). In addition, the enhanced immunomodulatory activity of SDH-0.2A, which contains →4)-α-D-GalpA-(1 → linkages, has led to speculation that it may involve TLR4 engagemen (63); however, direct structural evidence confirming the RGP-TLR4 interaction is still lacking.

Furthermore, the effect of processing (Paozhi) on glycosidic linkages, such as the increase in arabinose content and simplification of branching in SDP50-2 is observed to enhance bioactivity, but the underlying mechanism is not proven (11). Future work should integrate advanced techniques like molecular dynamics simulations to model linkage-specific interactions with biological targets.

### 4.4 Chemical modification to enhance function

The biological activity of native RGPs can be optimized through targeted structural modifications, which aim to overcome inherent limitations such as poor aqueous solubility, limited bioavailability, and lack of target specificity. Key chemical derivatization methods include sulfation, phosphorylation, carboxymethylation are employed to enhance RGP functionality, but the SAR underlying these modifications is often empirical. For example, acetylated RGPs show improved antioxidant activity (75), yet the relationship between the degree of substitution and bioactivity is not quantitatively established, and the mechanisms (e.g., changes in hydrophobicity or receptor affinity) are speculative. Beyond traditional chemical groups, nanotechnology offers a powerful approach to modify delivery and efficacy. Huang and colleagues (76) developed a PEGylated nano-RGP (pRL) via thin film hydration and ultrasonication, optimizing the formulation using RSM to achieve a lipid: cholesterol ratio of 8:1, 9% DSPE-PEG2000, and a 5.4:1 lipid: drug mass ratio. The resulting nanoparticles were characterized by a small size (31.98 ± 2.6 nm), high homogeneity (PDI = 0.208), and potentiated immunomodulatory effects on macrophages. The nano-formulation was internalized via macropinocytosis and caveolae-mediated pathways, and it significantly stimulated macrophage proliferation, pro-inflammatory cytokine release, and M1 polarization, highlighting its promise as an immune adjuvant. The application of RSM created a robust model linking process parameters to product quality, providing a basis for future standardization.

Phosphorylation introduces phosphate groups to enhance hydrophilicity and impart novel bioactivities (77). Liu et al. (78) synthesized phosphorylated RGP (P-RPS) with a mixed phosphate system, determining optimal conditions (70 °C, 104% phosphate, 7.2 h, 9.75% urea) through systematic optimization. P-RPS has demonstrated dual immunomodulatory and anti-gastric cancer activities, mediated through the downregulation of LGR6 and inhibition of the Wnt/*β*-catenin pathway. However, this mechanistic link has been inferred primarily from indirect evidence and requires direct experimental validation.

In summary, while the SAR discussion provides a foundational overview, it is imperative to acknowledge that many relationships remain qualitative and speculative. Contradictory findings across studies underscore the need for standardized materials and methods. Unresolved mechanisms, particularly regarding target engagement and signaling pathways, limit predictive capability. The evidence for microbiota-mediated effects, for instance, is primarily from animal models, and human data are lacking. Moving forward, a critical, mechanism-driven approach integrated with computational modeling and targeted experiments is essential to transform descriptive correlations into predictive SAR models.

## 5 Pharmacological properties

**Figure 3:**
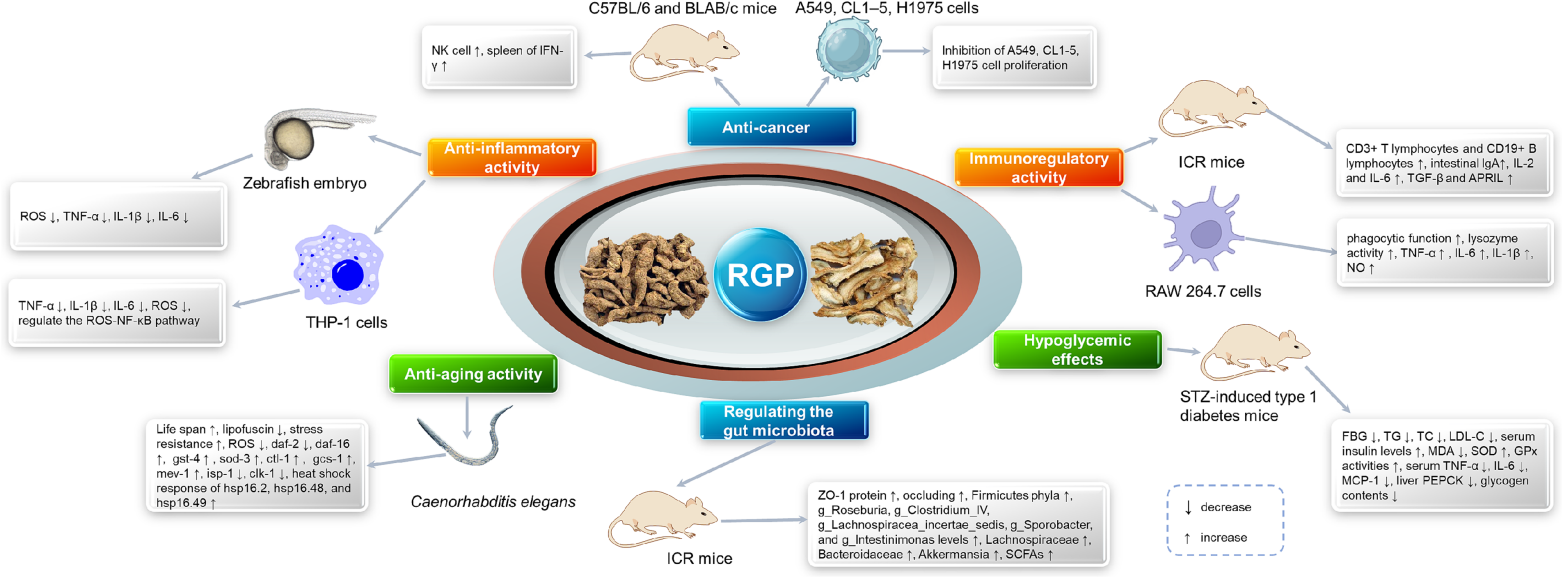
The pharmacological activities of *Rehmannia glutinosa* polysaccharides. The diagram visually integrates the multifaceted actions of RGPs, including immunomodulation, anti-inflammation, anti-aging, and metabolic regulation as presented in Section 5, emphasizing the interconnectivity between gut microecology, immune signaling, and systemic health outcomes.

Extensive *in vitro* and *in vivo* studies have established polysaccharides as the principal bioactive constituents responsible for the therapeutic effects of *Rehmannia glutinosa*. These RGPs exhibit a broad and promising spectrum of pharmacological properties, including immunomodulation, anti-inflammatory, antitumor, anti-aging, and metabolic regulatory activities, many of which are mediated through gut microbiota modulation. The following sections will detail these key activities, and a consolidated overview is provided in Table 3 and Figure 3.

**Table 3:** Biological activities of *Rehmannia glutinosa* polysaccharides and their underlying mechanisms of actions.

Biological activities	Polysaccharide name	*In vitro* or *in vivo*	Indicated concentrations	Models/Test system	Action or mechanism	References
Anti-cancer	RG-B9	*In vitro*	0–800 μg/mL	A549, CL1–5, H1975 cells	Inhibition of A549, CL1-5, H1975 cell proliferation	Lu et al. (116)
Anti-cancer	RGP	*In vivo*	50 mg/kg	C57BL/6 and BLAB/c mice	NK cell (+), spleen of IFN-*γ* (+)	Xu et al. (80)
Immunomodulatory effect	RGP	*In vivo*	100, 200, and 400 mg/kg	ICR mice	CD3^+^ T cells and CD19^+^ B cells (+), intestinal IgA (+), IL-2 and IL-6 (+), TGF-β and APRIL gene expression (+)	Yu et al. (96)
Immunomodulatory effect	SDH-WA	*In vitro*	1, 10, 20, 50 and 100 μg/mL	RAW264.7 cells	Macrophage phagocytic function (+), lysozyme activity (+), TNF-α (+), IL-6 (+), IL-1β (+), NO (+)	Zhou et al. (63)
Immunomodulatory effect	SDH-0.2A	*In vitro*	1, 10, 20, 50 and 100 μg/mL	RAW264.7 cells	Macrophage phagocytic function (+), lysozyme activity (+), TNF-α level (+), IL-6 level (+), IL-1β level (+), NO level (+)	Zhou et al. (63)
Immunomodulatory effect	RGLP	*In vitro*	0.3125–5.0 mg/mL	RAW 264.7 cells	TNF-α level (+),、IL-1β level (+)、IL-6 level (+), NO level (+)	Wang et al. (34)
Immunomodulatory effect	FRP	*In vitro*	12.5, 25, 50, 100, 200, 400 and 600 μg/mL	RAW 264.7 cells	TNF-α level (+),、IL-6 level (+)、IL-4 level (+), IL-10 level (+)	Qian et al. (58)
Immunomodulatory effect	DRP	*In vitro*	12.5, 25, 50, 100, 200, 400 and 600 μg/mL	RAW 264.7 cells	TNF-α level (+),、IL-6 level (+)、IL-4 level (+), IL-10 level (+)	Qian et al. (58)
Immunomodulatory effect	HRP	*In vitro*	12.5, 25, 50, 100, 200, 400 and 600 μg/mL	RAW 264.7 cells	TNF-α level (+),、IL-6 level (+)、IL-4 level (+), IL-10 level (+)	Qian et al. (58)
Immunomodulatory effect	NRP	*In vitro*	12.5, 25, 50, 100, 200, 400 and 600 μg/mL	RAW 264.7 cells	TNF-α level (+),、IL-6 level (+)、IL-4 level (+), IL-10 level (+)	Qian et al. (58)
Anti-aging activity	RGP50-2	*In vivo*	500 μg/mL	*Caenorhabditis elegans*	Life span (+), lipofuscin (−), stress resistance (+), ROS (−)	Shi et al. (11)
Anti-aging activity	SDP50-2	*In vivo*	500 μg/mL	*Caenorhabditis elegans*	Life span (+), lipofuscin (−), stress resistance (+), ROS (−), daf-2 level (−), daf-16 level (+), genes gst-4, sod-3, ctl-1 and gcs-1 expression levels (+), mev-1 expression levels (+), isp-1 and clk-1 expression levels (−), heat shock response of hsp16.2, hsp16.48, and hsp16.49 (+)	Shi et al. (11)
Anti-aging activity	RG70	*In vivo*	0.2, 0.4 and 0.8 mg/mL	*Caenorhabditis elegans*	Life span (+), lipofuscin (−), daf-16 level (+), skn-1 level (+), sod-3 and gcs-1 expression levels (+)	Liang et al. (72)
Hypoglycemic effects	RGP	*In vivo*	20, 40, 80 mg/kg	Male Kunming mice	FBG level (−), TG level (−), TC level (−), LDL-C level (−), serum insulin levels (+), MDA levels (−), SOD and GPx activities (+), serum TNF-α levels (−), IL-6 levels (−), MCP-1 levels (−), liver PEPCK mRNA expression (−), glycogen contents (−)	Zhou et al. (117)
Regulating the microbiota	RGP	*In vivo*	100, 200, and 400 mg/kg	Female ICR mice	ZO-1 protein levels (+), occluding levels (+), *Firmicutes phyla* (+), *g_Roseburia*, *g_Clostridium_IV*, *g_Lachnospiracea_incertae_sedis*, *g_Sporobacter*, and *g_Intestinimonas* levels (+), *Lachnospiraceae*, *Bacteroidaceae*, and *Akkermansia* (+), SCFAs (butyrat)	Yu et al. (96)
Regulating the microbiota	RGP	*In vivo*	400 mg/kg	Male ICR mice	Bacteroidetes and Firmicutes (+), Proteobacteria (+), Lactobacillus, Alistipes and Lachnospiraceae_NK4A13 (+), SCFAs (acetic acid, propionic acid and butyric acid) (+),	Lv et al. (92)
Anti-inflammatory effects	RGP70–2	*In vivo* and *In vitro*	25, 50, and 100 μg/mL	Zebrafish embryo, THP-1 cells	zebrafish embryos of ROS (−), TNF-α level (−), IL-1β level (−), IL-6 level (−); THP-1 cells of TNF-α level (−), IL-1β level (−), IL-6 level (−), ROS (−), regulate the ROS-NF-κB pathway	Zhang et al. (73)

(+): improve or promote. (−): inhibit or reduce.

### 5.1 Antitumor activity

Although research on the antitumor activity of RGPs is less extensive compared to other polysaccharides, existing studies have demonstrated their inhibitory effects on various tumor models. The primary mechanism underlying this activity appears to be the indirect eradication of cancer cells via potent immunomodulation. RGPs can activate both innate and adaptive immune responses; for instance, they induce dendritic cell maturation to enhance antigen presentation and T-cell activation (79), while concurrently stimulating the cytotoxic activity of natural killer (NK) cells and the secretion of antitumor cytokines like IFN-*γ* (76). This immunostimulatory effect creates a hostile microenvironment for tumors, effectively inhibiting growth and metastasis. Supporting this immunocentric view, one study confirmed that an RGP fraction elicited antitumor effects specifically by activating NK cells (80). In addition to immune-mediated mechanisms, RGPs may also exert direct effects on tumor cells. Evidence suggests they can directly induce apoptosis and cell cycle arrest in specific cancer lines, such as human chronic myeloid leukemia K562 cells (81). A potential molecular pathway for this direct action involves the upregulation of the tumor suppressor gene p53, which can trigger cell cycle arrest or apoptosis (82). However, the specific molecular pathways mediating these direct pro-apoptotic effects are not yet fully elucidated. Further research is needed to clarify the relative contributions and interplay between indirect immunomodulation and direct cytotoxicity in the overall antitumor activity of RGPs.

### 5.2 Immunomodulatory effects

Immunomodulation is a cornerstone of the pharmacological profile of RGPs. Substantial evidence indicates that a primary mechanism for this activity is the activation of the TLR4 signaling pathway in immune cells. This TLR4 engagement triggers a cascade that promotes the maturation and functional enhancement of antigen-presenting cells, particularly dendritic cells (DCs), which subsequently orchestrate adaptive immune responses, underpinning RGP’s immunostimulatory and anti-tumor potential.

A key immunomodulatory action of RGPs is the activation and maturation of DCs, which serve as critical bridges between innate and adaptive immunity. Research shows RGPs stimulate the maturation of bone marrow-derived and splenic DCs, markedly upregulating the surface expression of co-stimulatory molecules (CD80, CD86) and MHC-II (83–85). This potentiation of their antigen-presenting function is coupled with elevated secretion of cytokines like IL-12, which drives the differentiation of naïve T cells into Th1 cells. Consequently, RGP-activated DCs exhibit a significantly enhanced capacity to stimulate the proliferation of antigen-specific T cells.

The systemic immunorestorative effects of RGPs have been validated in immunosuppressed animal models. In cyclophosphamide (CTX)-treated mice, RGPs administration dose-dependently restored spleen and thymus indices, normalized peripheral blood cell counts, and stimulated the proliferation of B cells and T cell subsets (CD4^+^, CD8^+^) (86). Furthermore, RGPs treatment modulated intestinal cytokine levels, counteracting CTX-induced imbalances by reducing pro-inflammatory IL-6 and TNF-*α* while elevating anti-inflammatory IL-10, thereby alleviating inflammation and aiding immune recovery. Similarly, Jia et al. (87) demonstrated that an RGPs from steam-pressurized Rehmannia restored immune function in immunosuppressed mice, evidenced by increased organ indices, enhanced macrophage phagocytosis, and a balanced cytokine profile.

Notably, the immunomodulatory benefits of RGPs extend beyond mammalian systems, showing promise in aquaculture. Wu et al. (88) demonstrated that dietary supplementation with polysaccharides from processed Rehmannia (RRPP) significantly enhanced immune function in *Luciobarbus capito*. During a 60-day feeding trial, RRPP improved non-specific immune parameters dose-dependently (0.05–0.4%). The most effective dose (0.2%) significantly increased serum lysozyme, acid phosphatase, superoxide dismutase, and total protein activities (*p* < 0.05). RRPP also modulated immune-related gene expression, upregulating pro-inflammatory cytokines (IL-1*β*, IL-8, TNF-α, IFN-*γ*) and downregulating anti-inflammatory cytokines (IL-10, TGF-β) in immune-relevant tissues, suggesting a role in fine-tuning inflammatory balance. Critically, this immunostimulation translated to practical benefit, as the RRPP-supplemented group exhibited a markedly higher survival rate (63.4% vs. 23.3% in controls) following a bacterial challenge, highlighting its potential as an effective immunostimulant in aquaculture.

### 5.3 Anti-aging effects

RGPs have emerged as promising anti-aging agents due to their multi-target activities and favorable safety profile. The anti-aging efficacy of RGPs has been primarily assessed using established preclinical models, with the nematode *Caenorhabditis elegans* serving as a key model organism. For instance, a neutral polysaccharide from Rehmannia (NPRG) has been shown to significantly extend the lifespan of *C. elegansand* improve its tolerance to thermal and oxidative stress, providing direct evidence for RGP’s anti-aging potential (89). At the molecular level, RGPs are proposed to modulate several evolutionarily conserved longevity pathways. A well-established mechanism involves the insulin/IGF-1 signaling (IIS) pathway. In *C. elegans*, NPRG extends lifespan by inhibiting the IIS receptor homolog daf-2, leading to the nuclear localization of the FOXO transcription factor homolog daf-16and the subsequent upregulation of cytoprotective genes. Beyond IIS, other pathways are implicated. The synergistic Sirt1/Nrf2 axis, a central regulator of cellular stress resistance and metabolism, represents a plausible but unverified target for RGP action, as suggested by studies on other plant polysaccharides. Similarly, modulation of the AMPK/mTOR signaling network, a critical nutrient-sensing hub, is a recognized pro-longevity strategy. While direct evidence for RGP’s action on AMPK/mTOR is lacking, the fact that other Rehmannia components and plant polysaccharides modulate this pathway strongly warrants its investigation for RGPs.

An emerging and significant mechanism involves the gut microbiota. Polysaccharides like RGPs can be fermented by gut microbes into SCFAs, which contribute to gut health, systemic immunity, and energy homeostasis. Supporting this, Liang and colleagues reported that an RGP extended lifespan in *C. elegans* by altering the gut microbiota and metabolome, introducing the gut-brain axis as a novel mechanism for its anti-aging effects (72).

In summary, current evidence suggests RGPs combat aging through multiple, potentially interconnected mechanisms, including modulation of conserved longevity pathways (e.g., IIS) and via gut microbiota-mediated effects. Further research is needed to fully elucidate the contributions of pathways like Sirt1/Nrf2 and AMPK/mTOR, and to validate these findings in mammalian models.

### 5.4 Modulation of gut microecology

The gut microbiota serves as a critical interface between the host and the external environment. With aging, it undergoes profound dysbiosis characterized by a loss of diversity, a reduction in beneficial commensals, and an expansion of pathobionts. This age-associated microbial disturbance is a major contributor to immunosenescence and inflammaging, largely due to compromised intestinal barrier integrity and shifts in the microbial metabolome. Accumulating evidence identifies RGPs as effective prebiotic agents that can mitigate this dysbiosis and help re-establish gut and systemic immune equilibrium.

As macromolecules resistant to host digestion, RGPs primarily act in the intestine, where they serve as fermentable substrates for the gut microbiota. Their fermentation significantly increases the concentrations of SCFAs primarily acetate, propionate, and butyrate. SCFAs act not only as local regulators of the intestinal environment but also influence distant organs via systemic circulation, serving as a key bridge linking the gut microecology to systemic immune and metabolic homeostasis (90, 91). Supporting this, a growing body of research demonstrates that RGP-mediated health benefits are closely linked to SCFA production.

In inflammatory conditions such as colitis, RGPs have been shown to restore microbial balance and barrier function. For instance, in a DSS-induced colitis model, RGP administration significantly ameliorated disease symptoms. The core mechanism involved remodeling the gut microbiota by increasing the relative abundance of beneficial bacteria such as *Akkermansia muciniphilaand Lactobacillus*, while restoring SCFA levels (92). Similarly, a multi-omics study in a DSS-colitis model confirmed that RGP increased microbial *α*-diversity, elevated the *Firmicutes*/*Bacteroidetesratio*, enriched SCFA-producing genera (*Lactobacillus*, *Akkermansia*, *Bifidobacterium*), and suppressed pro-inflammatory bacteria like Enterobacteriaceae (93). Importantly, the promoted *Lactobacillusand Akkermansia* abundances showed a significant positive correlation with butyrate concentration. Butyrate, as the preferred energy source for colonocytes, helps reduce oxygen tension, creating an anaerobic environment that further suppresses the expansion of facultative anaerobic pathogens.

This microbiota-modulating effect of RGPs and the consequent SCFA production are conserved across species and are central to their systemic immunomodulatory action. Research in a carp model confirmed that RGP as a feed additive selectively promoted *Akkermansia* growth and suppressed the conditional pathogen Aeromonas (94). Furthermore, a mechanistic study delineated a clear “microbiota-SCFA-immunity” axis: Rehmannia polysaccharide (RP) boosted colonic SCFA levels, which subsequently activated the expression of their cognate receptors (FFAR2 and FFAR3) in colon tissue, thereby mediating immunomodulation (95). This pathway is particularly relevant in aging, as age-related dysbiosis leads to reduced SCFA production, exacerbating immunosenescence. Consistent with this, RGP intervention in aged models has been shown to increase fecal SCFA levels and improve intestinal barrier integrity (96). Although studies on specific bacterial strains capable of degrading RGPs are currently limited, potential key degraders may be inferred based on structural similarities. Genera such as *Bacteroides* (especially *B. thetaiotaomicron*), *Roseburia*, and *Bifidobacterium* possess genomes enriched with polysaccharide utilization loci (PULs) encoding glycoside hydrolases (GHs). For example, enzymes from the GH36 and GH3 families in *Roseburia intestinaliscan* cleave *β*-galactosidic bonds, which suggests that suggesting the galactose side chains of RGPs are likely a primary fermentation target (97). In summary, RGPs function as potent prebiotics that reshape the gut microbiota, enhance SCFA production, and activate downstream receptor-mediated signaling, thereby restoring gut ecological balance and exerting systemic anti-inflammatory and immunomodulatory effects.

While the aforementioned studies collectively support the prebiotic potential of RGPs, a critical evaluation of the reported microbial and immunomodulatory outcomes is warranted. The most robust and consistent findings across different animal models are: (1) the selective enrichment of specific beneficial genera, notably *Akkermansiaand* and *Lactobacillus*; and (2) the consequent increase in the production of SCFAs, particularly butyrate. Notably, these core effects appear largely independent of the specific disease context. However, variations in specific reported outcomes, such as the extent of *α*-diversity change, shifts in the *Firmicutes/Bacteroidetesratio*, or the spectrum of suppressed pathogens are commonly observed. These discrepancies can likely be attributed to several methodological factors: (i) Animal Model Differences: The baseline dysbiosis in a DSS-colitis model differs profoundly from that in an aging or healthy model, inherently shaping the observed compositional shifts. (ii) RGP Preparation and Dosage: Variations in extraction methods, Mw profiles, and administered doses between studies directly influence the magnitude and specificity of microbial modulation. (iii) Treatment Duration: The timing of sampling relative to the intervention period can capture either transient or stabilized microbial states. Therefore, while the general premise of an RGP-mediated microbiota-SCFA-immunity axis is well-supported, the precise taxonomic and metabolic details are model- and protocol-dependent. This context underscores that all current conclusions remain preliminary and are derived solely from preclinical evidence. Consequently, the imperative next step is to validate this conserved mechanistic axis in controlled human trials to determine its translatability and therapeutic relevance.

### 5.5 Metabolic disease management

RGPs demonstrate significant potential in managing metabolic disorders through multi-target mechanisms, with dysregulated glucose homeostasis and chronic low-grade inflammation being key intervention points. RGPs exert effective glycemic control via complementary mechanisms targeting both digestion and hormonal regulation. Chen and colleagues (46) isolated two RGP fractions (RGP70-1-1 and RGP70-1-2) that potently inhibited the carbohydrate-digesting enzymes *α*-glucosidase and α-amylase *in vitro*, a mechanism akin to the drug acarbose that slows postprandial glucose absorption. Notably, beyond enzyme inhibition, RGP70-1-1 significantly enhanced the secretion of the incretin hormone glucagon-like peptide-1 (GLP-1) from intestinal cells. This finding is crucial because GLP-1 potentiates glucose-dependent insulin secretion and promotes satiety, representing a major target in diabetes therapy. Mechanistically, the stimulation of GLP-1 secretion was associated with the activation of the PI3K/Akt signaling cascade, implying a potential role for RGPs in ameliorating insulin signaling and combating insulin resistance.

In addition to direct glycemic control, RGPs address the underlying chronic inflammation that drives metabolic disorder progression. The NF-κB pathway is a central regulator of such inflammatory responses. Research indicates that RGPs possess significant anti-inflammatory activity, with a core mechanism being the inhibition of NF-κB signaling. For instance, RGP intervention has been shown to downregulate the expression of Toll-like receptor 4 (TLR4) and NF-κB, thereby markedly inhibiting the production of pro-inflammatory cytokines like TNF-α, IL-6, and IL-1β (44). Given that these cytokines directly impair insulin signaling, the anti-inflammatory action of RGPs is integral to their overall metabolic benefits. This mechanism suggests that RGPs could improve insulin resistance by suppressing local inflammation in metabolic organs like adipose tissue and the liver, although this specific application within metabolic disease models warrants further direct investigation. In summary, RGPs manage metabolic disease through a dual approach: directly regulating glucose homeostasis via enzyme inhibition and incretin hormone stimulation, and indirectly improving metabolic function by mitigating the chronic inflammatory state that underlies insulin resistance.

### 5.6 Anti-inflammatory activity

Chronic and excessive inflammation underpins numerous diseases, driving the search for safe and effective therapeutic agents. RGPs have demonstrated promising anti-inflammatory properties, as evidenced by studies employing canonical inflammatory models. A widely utilized system for such evaluation is the LPS-induced macrophage activation model. LPS potently activates macrophages via TLR4, triggering a robust pro-inflammatory response.

Comprehensive research by Qiao et al. (98) evaluated RGP’s activity using an integrated approach encompassing in vitroRAW264.7 macrophages, an LPS-induced acute liver injury mouse model, and computational simulations. *In vitro*, RGPs treatment significantly suppressed LPS-induced intracellular ROS, apoptosis, and the secretion of NO, IL-6, IL-1β, and TNF-α. *In vivo*, RGPs ameliorated liver damage and reduced serum inflammatory markers. Mechanistic investigations pinpointed TLR4 as a direct target. Molecular docking and dynamics simulations revealed high-affinity, stable binding between RGPs and the TLR4/MD-2 complex. This binding functionally inhibits the downstream TLR4/MyD88/NF-κB signaling pathway, reducing NF-κB p65 nuclear translocation and blocking the inflammatory cascade.

Further supporting evidence comes from studies on specific RGP fractions. Zhang et al. (73) demonstrated that the fraction RGP70-2 effectively inhibited LPS-induced inflammation in zebrafish and THP-1 cells. Its mechanism also involved suppressing the ROS-NF-κB signaling axis, highlighting a consistent role for NF-κB pathway modulation. In summary, RGPs exert significant anti-inflammatory effects through multi-target mechanisms, with direct interference in the TLR4/NF-κB signaling pathway representing a central mode of action. These findings underscore the potential of RGPs as candidates for managing inflammation-associated conditions.

## 6 Novel polysaccharide constructs derived from *Rehmannia glutinosa* polysaccharide

### 6.1 Rehmannia polysaccharides in drug delivery systems

Polysaccharides, valued for their broad availability, structural diversity, excellent biocompatibility, and facile chemical modifiability, represent an ideal class of biomaterials for advanced drug delivery systems. However, natural polysaccharides like RGPs are frequently hampered by inherent drawbacks, including large molecular size, poor target specificity, and rapid systemic clearance, which limit their bioavailability and therapeutic effectiveness. To address these limitations, researchers have turned to nanodelivery technology, which can encapsulate drugs to improve pharmacokinetics, enable targeted delivery, and control release. Consequently, the primary research directions involve either using RGPs as a component of a delivery carrier or encapsulating it as an active agent within other nanocarriers.

A major focus has been the development of RGP-based liposomal systems to enhance its immunomodulatory action. Liposomes, as phospholipid bilayer vesicles, effectively encapsulate water-soluble polysaccharides, protecting them from degradation and altering their biodistribution. Huang and colleagues have systematically developed RGP-loaded liposomes (RGPL). In one study, they optimized preparation via reverse evaporation and response surface methodology, achieving an encapsulation efficiency of 72.75% (99). These RGPL nanoparticles significantly enhanced immunostimulatory activity, promoting splenic lymphocyte proliferation and, when used as an adjuvant for the PCV-2 vaccine, boosting IgG antibody production and macrophage function (84). Mechanistically, RGPL effectively promoted dendritic cell maturation, upregulating surface markers (MHC II, CD86) and increasing memory T cell populations (100). To further improve pharmacokinetics, polyethylene glycol (PEG) modification has been applied. Huang et al. (76) developed a PEGylated nano-RGP (pRL), which exhibited extended circulation and more effectively activated macrophages, promoting their polarization to the pro-inflammatory M1 phenotype. Building on this platform, subsequent work confirmed its potent vaccine adjuvant potential by enhancing pathogen-specific adaptive immunity (101). An even more advanced construct involved concentrically coating bacterial outer membrane vesicles (OMVs) onto the pRL nanoparticles. This pRL-OMV nanovaccine showed improved stability, immunogenicity, and efficient drainage to lymph nodes, activating broad immune signaling pathways (102).

Parallel to delivery system engineering, targeted chemical modification of RGP itself can enhance or confer novel bioactivities. For instance, phosphorylated RGP (P-RPS) activates M1 macrophage polarization, which indirectly suppresses gastric cancer cell proliferation, migration, stemness, and induces apoptosis, a mechanism linked to downregulation of LGR6 and inhibition of the Wnt/*β*-catenin pathway (78). Acetylated RGPs derivatives demonstrate significantly improved antioxidant and radical scavenging capacity compared to the native polymer (75). Synthesis of an RGP-iron (III) complex (RGP-Fe(III)) potentiated both antioxidant effects and anti-iron deficiency anemia activity (103).

### 6.2 Obstacles to clinical translation

Substantial preclinical data indicate the potential efficacy of RGPs against conditions such as type 2 diabetes, cancer, and autoimmune disorders, highlighting a promising translational outlook (46, 104). However, the translation of any candidate therapy requires definitive validation through phased clinical trials to establish its human efficacy and safety profile. A systematic search of clinical trial registries and academic databases revealed no registered human trials utilizing RGP as a defined, standalone intervention. In clinical practice, Rehmannia is typically employed as a key component in multi-herb formulas, such as Liuwei Dihuang Wan. While clinical studies on these formulas exist, they assess the integral effect of the whole prescription and cannot attribute benefits specifically to the polysaccharide fraction due to the immense analytical challenge of tracking individual components *in vivo* (105–107). Given this compelling preclinical evidence juxtaposed with a complete lack of clinical data, a critical question arises: what are the fundamental barriers hindering the clinical translation of RGPs? The translational impasse can be attributed to a confluence of scientific and practical challenges. (1) Structural Complexity and Unclear Quality Attributes: RGPs are not single chemical entities but heterogeneous mixtures. Their structural profile (Mw, monosaccharide composition, glycosidic linkages) varies significantly with geographical origin, processing method, and extraction protocols, making batch-to-batch standardization extremely difficult. (2) Poorly Defined SAR: The precise structural features responsible for specific pharmacological activities remain unclear. This limited understanding of SAR obstructs rational drug design and quality control based on bioactive motifs. (3) Inadequate Pharmacokinetic (PK) and Toxicological Profiling: As hydrophilic polymers, native high-molecular-weight RGPs exhibit very low oral bioavailability. Basic PK parameters, including the extent of absorption, identity of absorbed species, distribution, and elimination are virtually uncharacterized. Furthermore, while the long-term traditional use of Rehmannia suggests safety, comprehensive preclinical toxicological studies mandated for therapeutic development are largely absent for purified RGP extracts. (4) Manufacturing and Economic Hurdles: Developing a robust, scalable, and cost-effective Good Manufacturing Practice (GMP) process for high-purity, low-endotoxin RGP is technically challenging and requires substantial investment. Without proven clinical benefit, the economic rationale for such investment is weak. (5) Clinical Trial Design Complexity: RGP’s pleiotropic nature complicates clinical development strategy. Choosing a primary indication dictates trial design. Furthermore, its polypharmacology necessitates complex efficacy assessment using biomarker panels rather than a single endpoint, increasing trial complexity and cost (108). To bridge this translational gap, a strategic development pathway is required. Insights can be drawn from other plant polysaccharides that have advanced further in research (109–111). Key strategic imperatives include: (1) Prioritizing Indications: Focus on one or two indications where preclinical evidence is strongest, such as pre-diabetes or as an adjuvant in cancer immunotherapy. (2) Establishing Foundational Science: Dedicate resources to precise structural characterization, development of stability-indicating analytical methods, and conducting regulatory-grade PK and toxicology studies. (3) A pragmatic initial route could be developing a standardized RGP product as a functional food or dietary supplement. Post-marketing surveillance from such use could generate valuable human safety and preliminary efficacy data. (4) Pursuing a Targeted Research Agenda: Future research must focus on: (a) employing advanced analytics (multidimensional chromatography, MS, NMR) to build a comprehensive structural database and identify bioactive domains; (b) shifting from descriptive polypharmacology to precise mechanistic studies using tools like gene editing to pinpoint receptors and signaling pathways; (c) systematically investigating RGP’s interaction with the gut microbiota to elucidate how microbial shifts mediate systemic effects; and (d) innovating delivery systems (e.g., nanocarriers) to overcome poor bioavailability (112, 113). Only after substantial progress in these foundational areas should the initiation of meticulously designed clinical trials be contemplated.

Ultimately, these scientific and technical hurdles are compounded by significant regulatory and clinical trial challenges. The path to clinical approval for a complex botanical polysaccharide like RGPs is particularly demanding, requiring adherence to stringent regulatory standards for quality control and the successful navigation of phased clinical trials to demonstrate safety and efficacy in humans a process that is both time-consuming and capital-intensive.

## 7 Conclusion and perspectives

In conclusion, *Rehmannia glutinosa* polysaccharides (RGPs) represent a promising class of natural macromolecules with multifaceted pharmacological activities, particularly in immunomodulation and metabolic regulation. Their potential as therapeutics or functional food ingredients is, however, constrained by intrinsic challenges typical of plant polysaccharides: structural heterogeneity, elusive *in vivo* fate, and poorly defined structure–activity relationships. These interconnected bottlenecks hinder standardization, rational design, and clinical translation.

To transcend these limitations and unlock the translational potential of RGPs, future research must pivot from descriptive studies to a precision science paradigm. This necessitates converging efforts across multiple disciplines:

First, achieving structural precision is foundational. Moving beyond characterizing mixtures, the field must prioritize the complete elucidation of primary sequences and three-dimensional conformations of bioactive RGPs epitopes. This will require integrating advanced analytical technologies with computational modeling. Concurrently, exploring synthetic biology approaches to produce homogeneous, defined structures could revolutionize supply and eliminate batch variability.

Second, establishing predictive and mechanistic frameworks is crucial for rational development. Building comprehensive libraries of structurally defined RGPs fractions and correlating their features with biological outcomes through high-throughput screening and machine learning will enable the construction of quantitative structure–activity relationship (QSAR) models. Mechanistically, research must shift from observing polypharmacology to pinpointing primary molecular targets (e.g., specific immune receptors, gut microbial enzymes) and downstream signaling pathways, using genetic and molecular tools to validate causality.

Third, innovating targeted delivery and validating mechanisms in humans are key to clinical success. Advanced formulations, such as microbiota-responsive or ligand-decorated nanocarriers, should be engineered to overcome the poor bioavailability of RGPs and achieve site-specific action. Most importantly, the compelling preclinical evidence, especially regarding gut microbiota modulation and systemic immunity, must be rigorously tested in well-controlled human trials. Utilizing multi-omics approaches in these trials will be essential to validate the “microbiota-SCFA-immunity” axis in humans and identify biomarkers of response.

By embracing this interdisciplinary and precision-focused roadmap, RGPs research can evolve from empirical inquiry to the development of standardized, efficacious, and mechanism-based health products, ultimately bridging the gap between traditional herbal wisdom and modern evidence-based medicine.
